# Supplementary material for: Benchmarking Genomic Variant Calling Tools in Inbred Mouse Strains: Recommendations and Considerations
Source: bioRxiv. 2025 May 31:2025.05.28.656711. Preprint. [Version 1] doi: 10.1101/2025.05.28.656711 (PMC12154695; doi:10.1101/2025.05.28.656711)

bioRxiv preprint doi: https://doi.org/10.1101/2025.05.28.656711; this version posted May 31, 2025. The copyright holder for this preprint (which was not certified by peer review) is the author/funder, who has granted bioRxiv a license to display the preprint in perpetuity. It is made available under aCC-BY 4.0 International license.

Supplemental datasheet 1: Accession numbers and info for strain references
Supplemental datasheet 2: All metrics B6
Supplemental datasheet 3: All metrics diverse strains
Supplemental datasheet 4: Full model results for non-reference strains

Code documents on figshare

bioRxiv preprint doi: https://doi.org/10.1101/2025.05.28.656711; this version posted May 31, 2025. The copyright holder for this preprint (which was not certified by peer review) is the author/funder, who has granted bioRxiv a license to display the preprint in perpetuity. It is made available under aCC-BY 4.0 International license.

Supplemental Figure S1: Comparisons of SNP call sets across tools. (A) Upset plot of variant call sets at 10x coverage and allele balances 0.1, 0.5, and 0.9. Bar graphs show the intersection sizes between each combination of tools, with each colored bar showing the size of that set at the given allele balance. Set sizes to the left of the upset chart panel indicate the number of variants recovered by that tool under the simulated allele balance condition. (B,C,D) Classification tree predicting variant status based on the optimal combination of variant calling tools under different allele balance conditions. Simulated variants are represented as “1”, and false positives are indicated by “0”. Splitting criteria are displayed on the connecting branches, indicating the decision rule applied to divide the data. Terminal (leaf) nodes show the final predicted value and sample size for each group of observations. Node color intensity represents the relative probability that an observation in that node belongs to the predicted class, with green indicating a higher probability (closer to 1) and blue indicating a lower probability (closer to 0). Darker colors correspond to more confident predictions, while lighter colors indicate greater uncertainty or class mixing within the node.

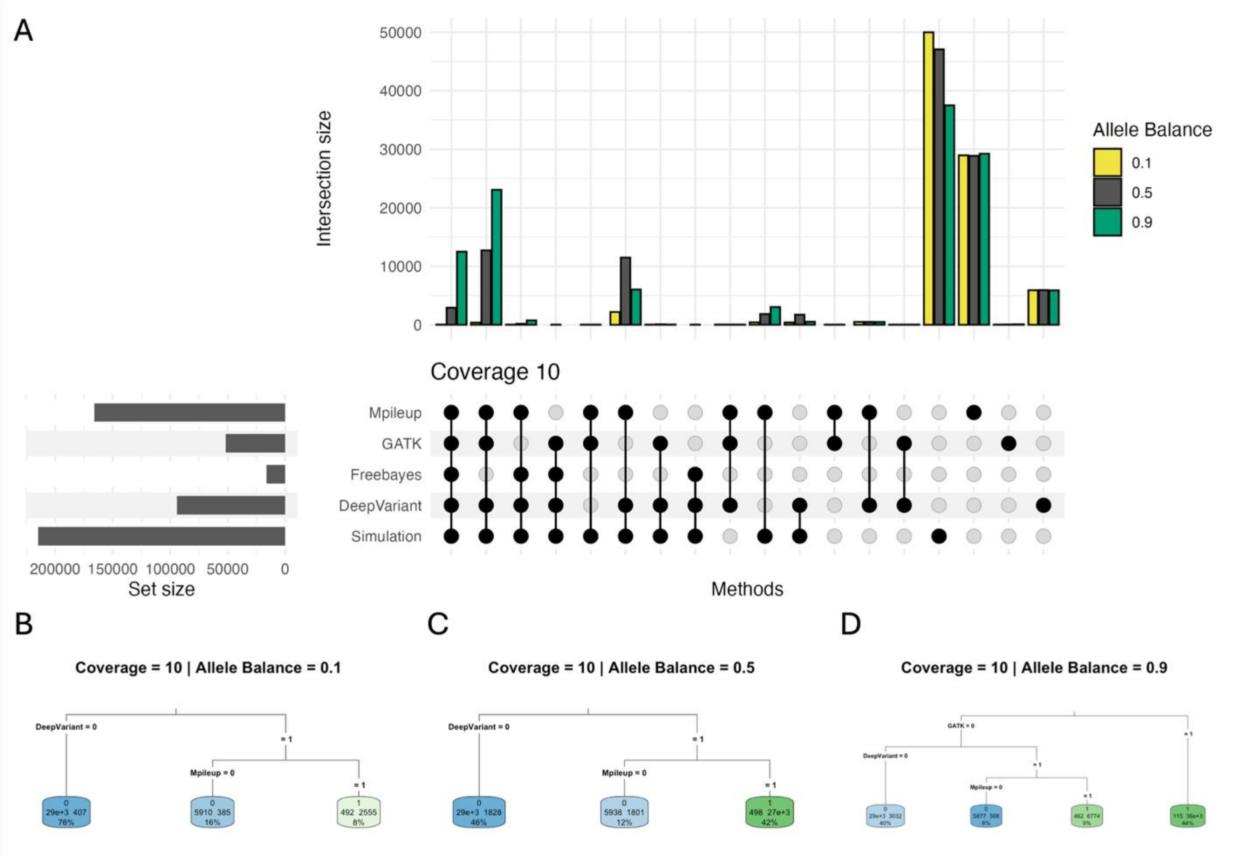

bioRxiv preprint doi: https://doi.org/10.1101/2025.05.28.656711; this version posted May 31, 2025. The copyright holder for this preprint (which was not certified by peer review) is the author/funder, who has granted bioRxiv a license to display the preprint in perpetuity. It is made available under aCC-BY 4.0 International license.

Supplemental Figure S2: Comparisons of SNP call sets across tools. (A) Upset plot of variant call sets at 40x coverage and allele balances 0.1, 0.5, and 0.9. Bar graphs show the intersection sizes between each combination of tools, with each colored bar showing the size of that set at the given allele balance. Set sizes to the left of the upset chart panel indicate the number of variants recovered by that tool under the simulated allele balance condition. (B,C,D) Classification tree predicting variant status based on the optimal combination of variant calling tools under different allele balance conditions. Simulated variants are represented as “1”, and false positives are indicated by “0”. Splitting criteria are displayed on the connecting branches, indicating the decision rule applied to divide the data. Terminal (leaf) nodes show the final predicted value and sample size for each group of observations. Node color intensity represents the relative probability that an observation in that node belongs to the predicted class, with green indicating a higher probability (closer to 1) and blue indicating a lower probability (closer to 0). Darker colors correspond to more confident predictions, while lighter colors indicate greater uncertainty or class mixing within the node.

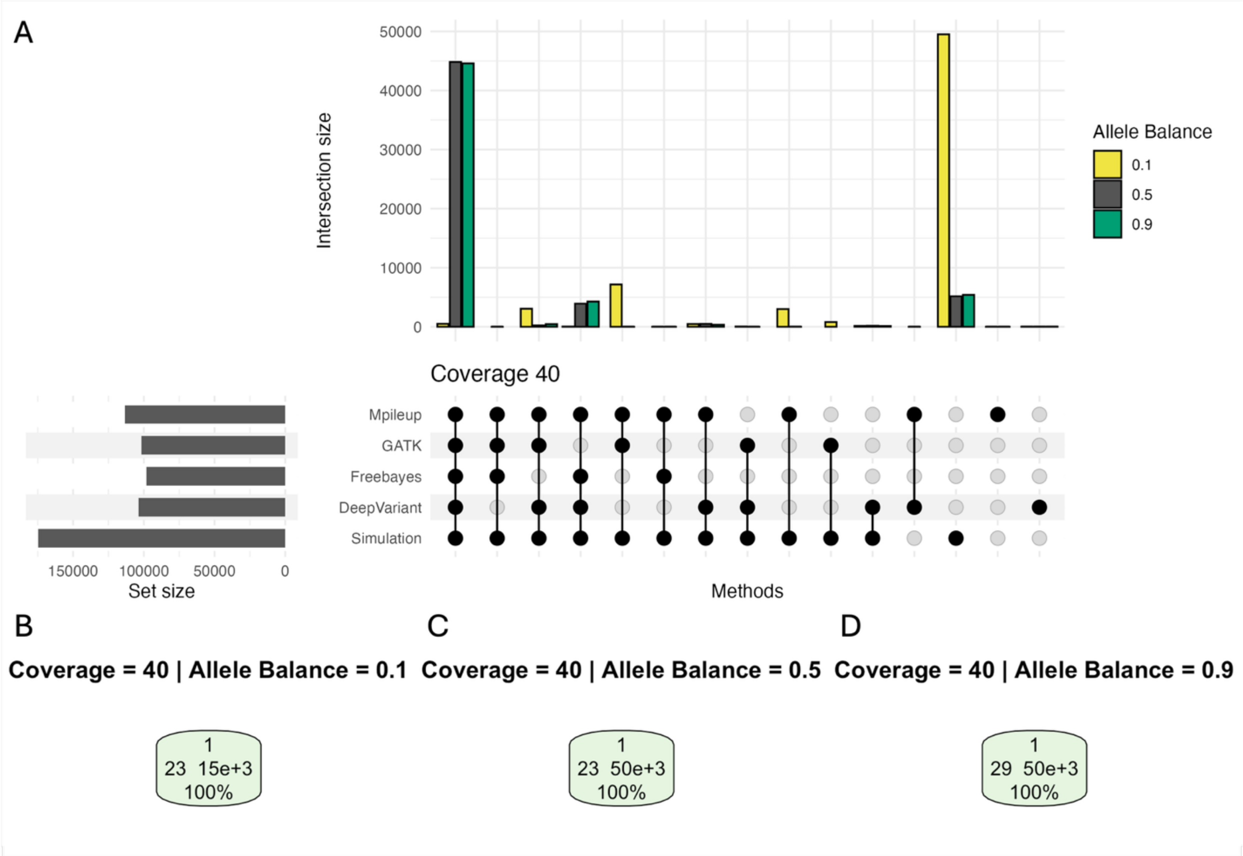

bioRxiv preprint doi: https://doi.org/10.1101/2025.05.28.656711; this version posted May 31, 2025. The copyright holder for this preprint (which was not certified by peer review) is the author/funder, who has granted bioRxiv a license to display the preprint in perpetuity. It is made available under aCC-BY 4.0 International license.

Supplemental Figure Set 3: All trees B6, all trees diverse strains.

Supplemental Figure S4. Post optimal filtration variant calling tool performance comparison across all allele balance and coverage values for Precision and Recall.

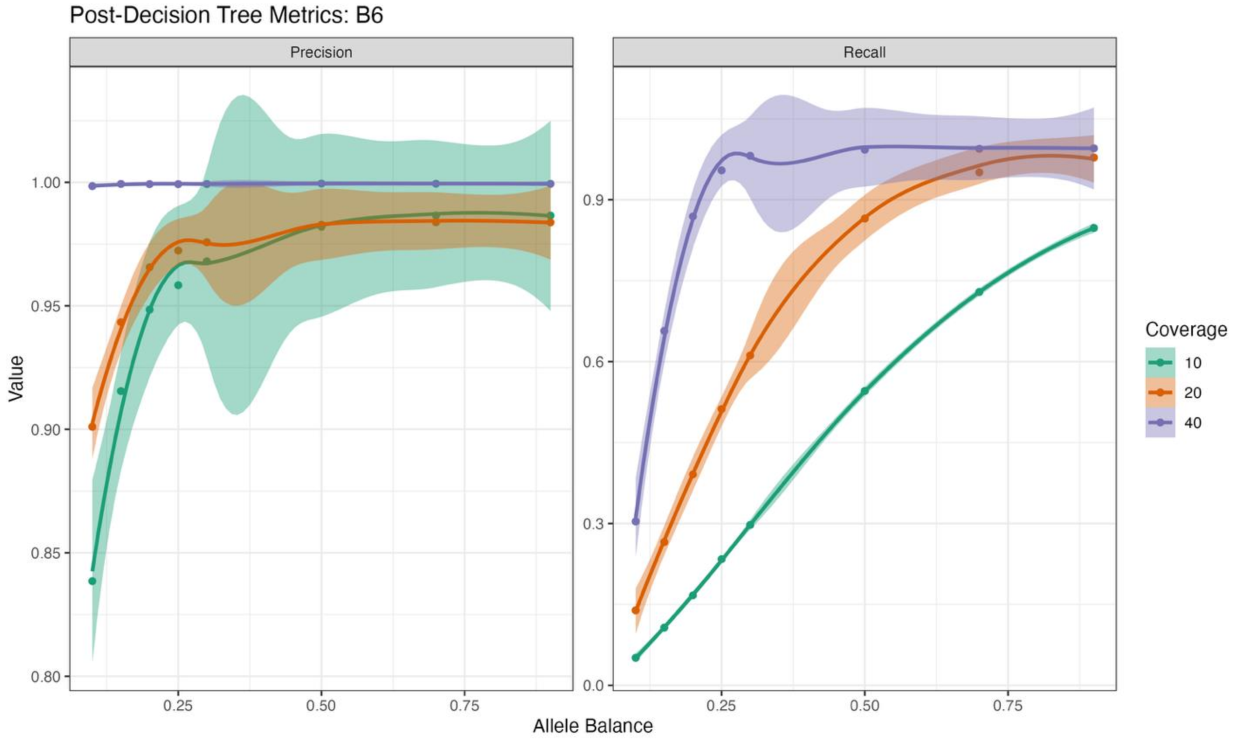

bioRxiv preprint doi: https://doi.org/10.1101/2025.05.28.656711; this version posted May 31, 2025. The copyright holder for this preprint (which was not certified by peer review) is the author/funder, who has granted bioRxiv a license to display the preprint in perpetuity. It is made available under aCC-BY 4.0 International license.

Supplemental Figure S5. Post optimal filtration variant calling tool performance comparison across all allele balance and strains by Precision, Recall, FP (the number of False Positive calls remaining in the optimal dataset), TP (the number of True Positive calls remaining after filtration), and FN (variants initially called correctly, but subsequently eliminated by the filtration approach).

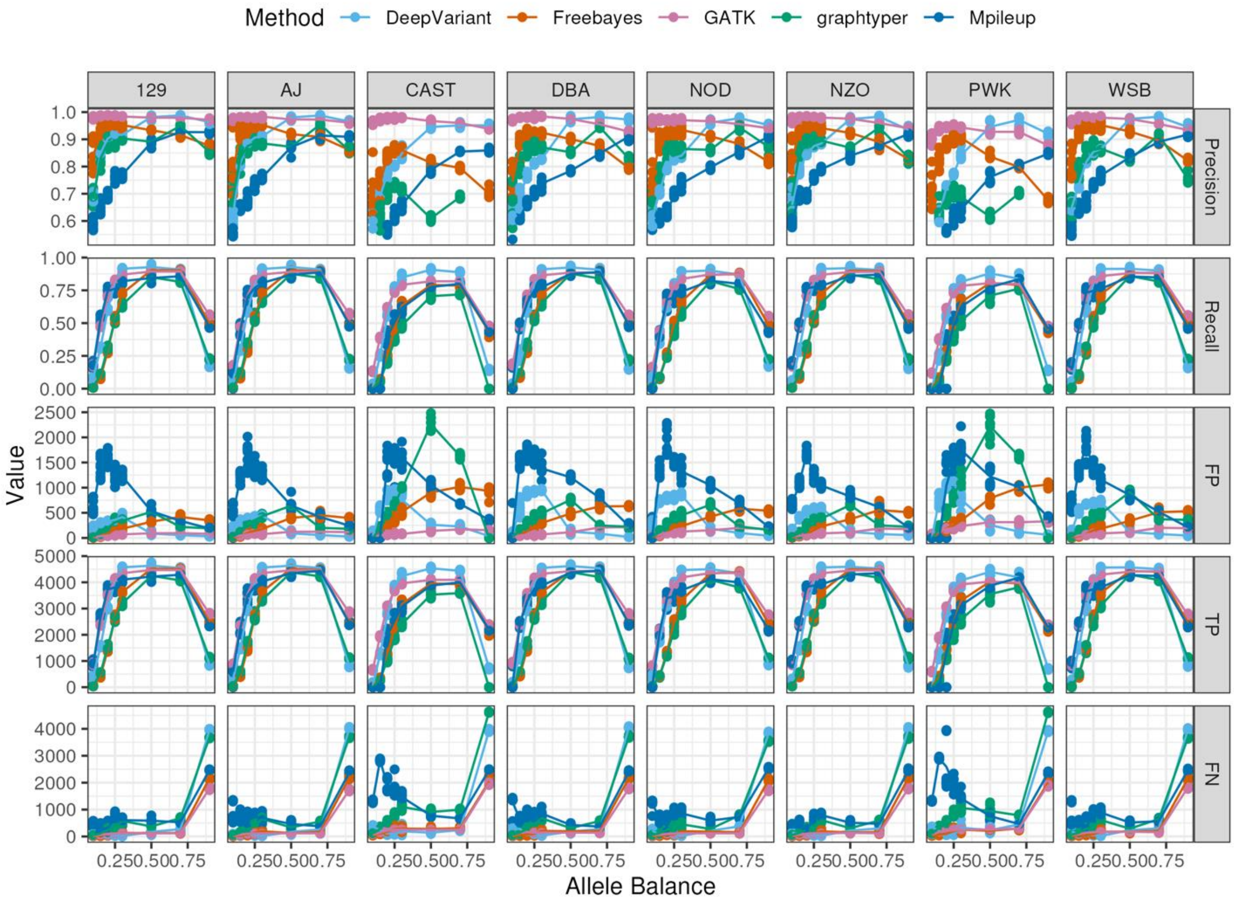

Supplement: 1 [file NIHPP2025.05.28.656711V1-supplement-1.pdf]
